# Post-Pandemic Insomnia in Healthcare Workers: A Prospective Study including Sociodemographic, Occupational and Psychosocial Variables

**DOI:** 10.3390/jcm13123498

**Published:** 2024-06-14

**Authors:** Fernanda Gil-Almagro, Francisco Javier Carmona-Monge, Fernando José García-Hedrera, Cecilia Peñacoba-Puente

**Affiliations:** 1 Psychology Department, Facultad Ciencias de la Salud, Universidad Rey Juan Carlos, Av. de Atenas, s/n, 28922 Madrid, Spain; fgilalmagro@gmail.com; 2 Nurse Intensive Care Unit, Hospital Universitario Fundación Alcorcón, Budapest, 1, 28922 Madrid, Spain; fjgarciah@gmail.com; 3 Anesthesia Department, Hospital Universitario Santiago de Compostela, Rúa da Choupana, s/n, 15706 A Coruña, Spain; javichun@gmail.com

**Keywords:** COVID-19 pandemic, insomnia, psychological resilience, social support, self-efficacy, cognitive fusion, evolution

## Abstract

**Background/Objectives**: Previous research points to the prevalence of insomnia during the COVID-19 pandemic among healthcare workers (HCWs). However, longitudinal studies on its evolution, including the post-pandemic stage, are less abundant, with a special lack of research about possible psychosocial risk factors. The aim of the current study is to analyze the evolution of insomnia in Spanish HCWs from the beginning of the pandemic until two years later, examining the influence of sociodemographic, occupational and psychosocial variables. **Methods**: This was a prospective longitudinal design with three data collection periods in which insomnia was assessed using the Insomnia Severity Index (ISI), in addition to sociodemographic, occupational and psychosocial (i.e., social support, self-efficacy, resilience and cognitive fusion) variables in HCWs (n = 216) who were in direct contact with COVID-19 patients. **Results**: High scores were observed for insomnia, with a clear decrease throughout the periods studied (F = 30.198, *p* < 0.001). An association was observed between insomnia and certain sociodemographic and occupational variables (i.e., work category, *p* = 0.001; availability of Personal Protective Equipment (PPE), *p* < 0.001; workload, *p* < 0.001; worry about self or family contagion, *p* = 0.002, *p* = 0.003, respectively). Insomnia had negative relationships with social support (*p* = 0.014), self-efficacy (*p* < 0.001) and resilience (*p* < 0.001) and positive relationships with cognitive fusion (*p* < 0.001). Interaction effects between the evolution of insomnia and social support from friends (*p* = 0.024, ƞ2 = 0.34) and between the evolution of insomnia and cognitive fusion (*p* = 0.047; ƞ2 = 0.25) were found. **Conclusions**: Social support, self-efficacy and resilience act as buffers for insomnia. Cognitive fusion acts as a clear precipitator of insomnia as well as directly influencing its evolution. Social support from friends also affects the evolution of insomnia. Within the specific circumstances of the COVID-19 pandemic, a long-term effect of social support from friends and a short-term effect of cognitive fusion on the evolution of insomnia was observed. The findings emphasize the need to implement specific interventions to promote the mental well-being of HCWs, particularly in crisis contexts that involve an increase in occupational stress, emphasizing the role of certain psychosocial variables as protective factors.

## 1. Introduction

Psychoemotional disturbances suffered by healthcare workers (HCWs) have been the subject of much research since the COVID-19 pandemic [1,2,3]. Some of the most interesting emotional consequences observed have been the alterations in sleep patterns, and several studies have been carried out to determine the prevalence of insomnia in HCWs during and after the pandemic [4,5]. Prior to the COVID-19 pandemic, insomnia was already a topic of interest in HCWs, with a generally high prevalence, and had been directly associated with night shifts or shift work [6]. Since the COVID-19 pandemic, an increased prevalence of this disorder has been identified as being related to high anxiety derived from bedside work with infectious patients or fear of contagion [4,5]. The most widely used instrument for its measurement has been the Insomnia Severity Index (ISI) [6].

During the COVID-19 pandemic, insomnia received special attention as a prevalent symptom in the general population and not only in HCWs. A systematic review, conducted between November 2019 and July 2021, included 250 studies from 49 different countries; it was found that during the COVID-19 pandemic, the estimated overall prevalence of sleep disturbances was 40.49% [95%CI 37.56–43.48%] [7], with the highest prevalence being among infected patients (52.39% [95%CI 41.69–62.88%]) and HCWs (42.47% [95%CI 37.95–47.12%]. Another review conducted during the pandemic, in 2021, compared insomnia between the general population and HCWs, reporting a higher prevalence among the latter [8].

Insomnia in HCWs is a severe occupational risk factor, and its especially high incidence among HCWs during the COVID-19 pandemic has prompted specific research into the matter. In particular, different studies indicate a high rate of insomnia among HCWs in the first months of the pandemic due to workload, lack of knowledge of the disease, fear of contagion and bedside work with infectious patients [7]. A review conducted in 2022 analyzing psychoemotional disturbances in HCWs, specifically insomnia [9], included 392 studies with single data and 18 studies with longitudinal data. The results showed elevated levels of insomnia for nurses (26.0% [95% CI 16.0–37.3]) and physicians (16.0% [95% CI 10.2–22.7]). A further review that included 18 studies between 2020 and 2021 analyzed the prevalence of different psychoemotional disorders suffered by HCWs during the COVID-19 pandemic, finding that although prevalence for all disorders was high, insomnia was the most elevated (anxiety 29.55%, depression 38.79%, post-traumatic stress disorder 29.8% and insomnia 40.66%) [10].

Most of the research in this area was carried out at the beginning of the COVID-19 pandemic, covering up to the summer of 2020. Data collected worldwide at that time reported high insomnia rates for the general population, ranging from 30% to 50% [8,9,10]. Other studies of a correlational nature analyzed the prevalence of insomnia years after the onset of the pandemic, finding that in HCWs, this high level of insomnia has remained, with a high percentage of nurses with sleep disorders in 2023 using the ISI instrument. In particular, it has been reported that 76.8% of nurses were still suffering from sleep disturbances three years after the start of the COVID-19 pandemic [11].

With regard to the countries under study, although insomnia research has been carried out in many countries, there is no doubt that China has been one of the most studied contexts, with the highest percentages of insomnia observed among HCWs [11]. More specifically, the prevalence of insomnia in China among HCWs was estimated at 40% [12] during 2021, although other studies reported even higher percentages of around 50% [13], with nurses as the most prevalent population compared to physicians [13].

In this context, given the severity of insomnia among HCWs, it is still of interest to study its prevalence after the pandemic, re-analyzing possible risk factors recorded previously, such as shift work [14]. However, over time, it seems as though the study of this alteration and its relationship with the COVID-19 pandemic has been neglected, although it is necessary to continue to follow up on HCWs in what has come to be called “the post-pandemic era of COVID-19”. It is essential to understand the real impact of the pandemic and the variables that may have facilitated the chronification of insomnia, once the acute stress stage had ended. Thus, longitudinal studies become particularly relevant to understanding the real evolution and consequences derived from a stressful work stage, such as the COVID-19 pandemic. Some research has been carried out to address this need; for example, in China, insomnia in nurses was analyzed during the peak of the pandemic and one year later, with higher means one year after the onset of the pandemic (6.90 [SD = 6.01] vs. 6.57 [SD = 5.64]) [15]. A longitudinal study in Italian nurses reported similar results as above, recording higher levels of insomnia for nurses during the second data collection period (peak vs. second wave of COVID-19) [16].

Given the above, there seems to be a need to gather longitudinal data regarding the prevalence of insomnia in HCWs since the beginning of the COVID-19 pandemic. Although the existing literature suggests that the evolution of anxiety derived from the pandemic on HCWs seems to be decreasing, registering lower levels of anxiety with the passage of time [17], the same does not seem to be true for insomnia. However, there are few longitudinal studies analyzing this in HCWs over the years [15,16].

Regarding the study of possible risk factors in the onset and maintenance of insomnia, previous research has focused mainly on the study of sociodemographic and occupational variables. Different studies of HCWs during the COVID-19 pandemic suggest that the fear of working with infectious patients, work overload or lack of Personal Protective Equipment (PPE) were all risk factors for insomnia in HCWs [18,19]. These studies have also found a higher prevalence of insomnia among nurses compared to physicians [20]. In addition to the above, shift work, including night shift rotation [21] and the development of care activities in highly complex units such as intensive care units (ICU) [13], has also been established as a risk factor.

In contrast to the interest in sociodemographic and occupational variables as risk factors for insomnia in HCWs, the study of the possible influence of psychosocial variables has received little attention and the findings are not entirely consistent. It has been suggested that low levels of resilience are related to higher means for anxiety; however, no association has been found between this personality trait and insomnia [22]. Meanwhile, other studies have found a clear negative relationship between resilience and insomnia [23]. Cognitive fusion has also been studied in HCWs, suggesting a clear relationship with anxiety [24]; however, its relationship with insomnia has not been studied either.

Given the paucity of research on the subject, it seems relevant to carry out prospective studies in the mid and long term, allowing us to understand the evolution of disorders such as insomnia—especially now that the most intense and complex phase of the COVID-19 pandemic is over. Trying to shed some light on this need, the aim of the current study is to analyze the evolution of insomnia, through the use of the ISI, in a sample of Spanish HCWs who in the first stage of the pandemic were in direct contact with infectious patients. The professionals were followed up, including two additional measures, at six months after the first stage of the pandemic and one year after the second time point. As an additional objective, the current study aims to analyze the possible association of insomnia and its evolution with sociodemographic, occupational and psychosocial variables.

In our opinion, our research presents some new and interesting issues compared to previous research: (1) It aims to analyze the prevalence of insomnia during the COVID-19 pandemic, through a prospective design with three data collection periods, with the aim of also investigating the so-called post-pandemic symptomatology. (2) In addition to the sociodemographic and occupational variables associated with insomnia, it specifically analyzes the associated psychosocial variables, which have hardly been studied in this regard. (3) With respect to the previous point, the influence of psychosocial variables is not only analyzed in a punctual manner at each point in time, but also in terms of their influence on the evolution of insomnia over the total time period considered.

## 2. Materials and Methods

**Figure 1:**
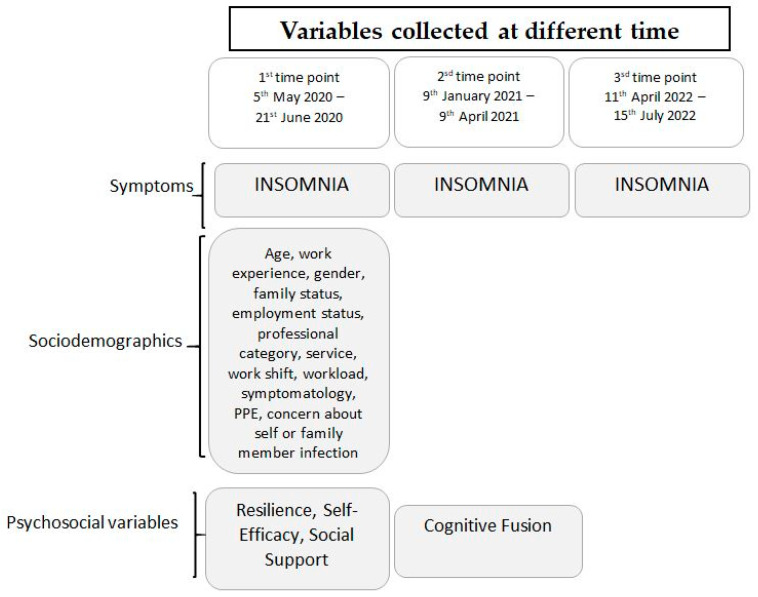
Prospective study design and variables involved.

Design: A prospective longitudinal study was conducted with three data collection periods: (1) (T1) between 5 May and 21 June 2020 (final phase of the state of alarm declared in Spain on 14 March), (2) (T2) six months after the end of the state of alarm (January–April 2021), and (3) (T3) one year after this second evaluation (April–July 2022). During the first data collection period, Spain was in a state of alarm involving a confinement phase. The state of alarm lasted until 21 June 2020. During the second data collection period, the situation was still complicated with 3,347,512 confirmed cases and 76,328 deaths as of 9 April 2021. The third data collection period ended with 12,973,615 confirmed COVID-19 cases and 108,730 deaths. During the three time periods, participants’ insomnia was assessed for its evolution. At the same time, different sociodemographic, occupational and psychosocial variables were evaluated during the first and second time points (see the Instruments section for more detailed information). Figure 1 shows the evaluation moments and the variables involved in the research.

Procedure and participants:

Data collection was carried out by means of an online electronic questionnaire designed for this purpose by the research team. At the beginning of the questionnaire, the main objective of the study was explained and informed consent was requested from all participants, as well as the explicit use of e-mail for contact in the successive evaluation phases of the research.

The sample was composed of HCWs from different services of the Spanish National Healthcare System. Probabilistic convenience sampling was carried out taking into account the following inclusion criteria: being an HCW (physician or nurse); carrying out their care activity within the different services of the Spanish National Healthcare System (both public and private); being 18 years of age or older; and having been in direct contact with COVID-19 patients. The exclusion criterion included having carried out healthcare activity in different services during the data collection period. A minimum sample size of n = 120 for prospective studies was taken as a reference [25]. However, taking into account the usual sample loss due to the longitudinal nature of the study and adding the complicated circumstances of the COVID-19 pandemic in HCWs [26,27], a minimum sample size of 720 participants was established for the first time point. Finally, at that first evaluation stage, we had a total of 1121 HCWs participating in this study. Of these, 403 HCWs continued to participate at the second time point, and 216 maintained their participation in the third evaluation time point, constituting the final sample of this study (n = 216).

To obtain the sample, the link with the questionnaire was sent to HCWs belonging to the Spanish National Healthcare System, both public and private. The questionnaire was distributed via social networks (Facebook, LinkedIn, Twitter and WhatsApp), as well as through the corporate e-mails of the different public and private services of the Spanish National Healthcare System. For the distribution of the questionnaire during the second and third time points, the e-mails of the HCWs who had participated in the first evaluation were used, requesting their participation again in the following phases of this study.

Variables and instruments:

- Insomnia [T1, T2, T3]:

The presence of insomnia symptoms was assessed using the Spanish version of the Insomnia Severity Index (ISI) [6,28]. This questionnaire measures insomnia briefly, following the criteria of the Diagnostic and Statistical Manual of Mental Disorders and the International Classification of Sleep Disorders. It is composed of 7 items that provide information on 3 factors (severity, impact and satisfaction). Each item is answered with a Likert-type scale from 0 (no problem) to 4 (many problems), obtaining a total score between 0 and 28, establishing the cut-off point at 22 for severe clinical insomnia. Cronbach’s alpha for this scale in the present study was 0.87.

- Sociodemographic and occupational variables [T1]:

An ad hoc questionnaire developed by the research team was used to collect these data. Specifically, we evaluated sociodemographic data (age, gender, personal situation) and occupational data (service in which they work, work experience in years, work shift (fixed, rotating or more than 10 h), availability of PPE, work overload (equal to usual, greater than usual)).

For the measurement of concerns about COVID-19, two ad hoc questions were designed to assess concerns about self-infection on the one hand, and concerns about infection of a family member on the other. Both items had a 4-point Likert-type response format (from 1 “not at all concerned” to 4 “very concerned”).

- Psychosocial variables [T1, T2].
- Social support [T1]: Social support was measured using the Multidimensional Scale of Perceived Social Support (MSPSS) instrument [29] in its Spanish version [30]. It consists of 12 items distributed in three dimensions: family, friends and significant others (4 items per dimension) on a 7-point Likert-type response scale from 1 = completely disagree to 7 = completely agree. An overall social support score is obtained by adding up the scores on the three subscales. The instrument has shown good psychometric properties in different studies [31,32]. Cronbach’s alpha for total social support in our study was 0.85. Its three dimensions also showed high internal consistency: social support family (0.81), social support friends (0.82) and social support from significant others (0.79).
- Self-efficacy [T1]: It was assessed through the General Self-Efficacy Scale (GSES) instrument [33] in its Spanish version [34]. This instrument is composed of 10 items on a 4-point Likert-type scale ranging from 1 “completely disagree” to 4 “completely agree”. The total score range is between 10 and 40. In our study, this instrument showed excellent internal consistency (α = 0.91).
- Resilience [T1]: The Spanish version of the Resilience Questionnaire (RS-14) was administered [35]. The RS-14 consists of 14 items with a 7-point Likert-type response format, from 1 (strongly disagree) to 7 (strongly agree), with a total scale score ranging from 14 to 98, with higher scores indicating greater resilience. Cronbach’s alpha in our study was 0.94.
- Cognitive fusion [T2]: The Cognitive Fusion Questionnaire (CFQ) was used [36] in its Spanish version [37], composed of 7 items assessing cognitive fusion (the extent to which we are psychologically entangled with or dominated by the form and content of our own thoughts). It is assessed with a Likert-type response scale with 7 alternatives, ranging from 1 (never) to 7 (always), with a total scale score ranging from 7 to 49, with higher scores reflecting a greater degree of cognitive fusion. Cronbach’s alpha was 0.97 in our study.

Data analysis: Descriptive analysis and Cronbach’s alpha were performed. Qualitative variables were described with frequencies (n) and percentages (%) and quantitative variables with mean (M) and standard deviation (SD). To analyze the bivariate associations between sociodemographic, occupational and psychosocial variables with insomnia, Student’s *t*-test, one-factor analysis of variance (ANOVA) and Pearson’s correlation were used, depending on the nature of the variables analyzed. For the assessment of the evolution of insomnia, a repeated measures analysis was carried out using the general linear model. Finally, for the evaluation of the influence of the psychosocial variables considered in the evolution of insomnia, mixed models within the general linear models of repeated measures were used, using the psychosocial variable as an inter-subject factor. Statistical analysis was performed with the Statistical Package for the Social Sciences (SPSS), version 21 for Windows. The results were considered statistically significant for values of *p* < 0.05.

## 3. Results

### 3.1. Characteristics of the Sample

Table 1 shows the sociodemographic and occupational characteristics of the participants. The sample was composed of 216 HCWs, 151 nurses (69.9%) and 65 physicians (30.1%), with a mean age of 42.89 years (SD = 9.84) and an age range between 21 and 66. The majority of the sample was composed of women (n = 182, 84.3%) and most of them were married or had a stable partner (n = 150, 69.4%). In terms of distribution by service, the sample was especially concentrated in the ICU (n = 77, 35.6%) and hospitalization (n = 59, 27.4%). The participants had a mean number of years of experience of 11.06 (SD = 9.29; range 0–35). When analyzing employment situation, half of the participants were permanent (n = 114, 52.8%). Most of the HCWs reported a greater than usual work overload (n = 180, 83.3%), in addition to a lack of availability of PPE (n = 126, 58.4%). Concerns about infecting a family member (Mean = 3.54, SD = 0.84) were higher than concerns about self-contagion. (Mean = 2.70, SD = 0.89).

### 3.2. Relationship of Insomnia Assessed at Each of the Three Time Points with Sociodemographic and Occupational Variables

Table 1 shows the associations between insomnia at the three different time points and the sociodemographic, occupational and COVID-19 variables (the latter assessed at T1). Significant associations of insomnia were observed with job category, family situation, perceived workload, availability of PPE, and concerns about self-contagion or other family members being infected.

As can be seen in Table 1, nursing professionals presented higher insomnia scores than physicians at all time points, being statistically significant in T1 (*p* = 0.001) and in T2 (*p* = 0.038), with these differences disappearing later. HCWs who did not have PPE availability showed higher levels of insomnia at T1 (*p* < 0.001), with this difference being diluted at the rest of the assessments.

The higher than usual workload showed a significant association with insomnia levels, with our study having found that HCWs who reported having a higher than usual workload had higher levels of insomnia. This difference remained significant at all three time points (*p* < 0.001, *p* < 0.001, *p* = 0.040).

HCWs with a partner had higher insomnia scores than those without a partner at T1 (*p* = 0.043). Worry about self or family member contagion had a significant positive correlation with insomnia assessed at all three time points in this study (all *p* < 0.01).

### 3.3. Evolution of Insomnia

At T1, a mean score of 11.72 points on the ISI scale (SD = 5.96) was observed, while at T2, the mean score was of 10.32 (SD = 6.22) and finally, at T3, a score of 9.76 (SD = 6.08) was observed. Based on the classification established by the ISI [6], Table 2 shows the distribution of insomnia levels at the three time points.

In T1, the percentage of professionals with subclinical insomnia or moderate insomnia was relatively high (n = 90, 41.7%; n = 59, 27.3%, respectively); furthermore, a small part of the sampled participants also reported suffering from severe insomnia (n = 13, 6.0%). Nurses and physicians reporting subclinical or moderate insomnia were 89 (41.2%) and 45 (20.8%), respectively, at T2. By T3, the number of professionals who reported suffering from subclinical insomnia (n = 90, 41.7%) or moderate insomnia (n = 44, 20.4%) remained the same, although a slight decrease was observed in the professionals who reported suffering from severe clinical insomnia (n = 5, 2.3%).

**Figure 2:**
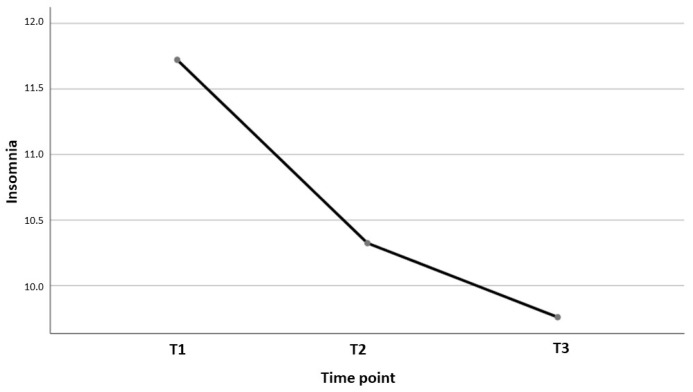
Evolution of insomnia at the three points in time.

The decrease in scores over time was statistically significant (F = 30.198, *p* < 0.001), and with a partial eta squared value of 0.123. Specifically, differences in insomnia levels were found between T1 and T2 (t(215) = 4.553, *p* < 0.001) and between T1 and T3 (t(215) = 5.495, *p* < 0.001), these not being significant between T2 and T3 (t(215) = 1.535, *p* = 0.126). Figure 2 shows the evolution of insomnia at the three time points.

### 3.4. Evolution of Insomnia as a Function of Social Support, Self-Efficacy, Resilience and Cognitive Fusion

#### 3.4.1. Descriptive Data on the Psychosocial Variables and Their Relationship with Insomnia Evaluated at the Three Time Points

As shown in Table 3, significant negative associations of social support in all its dimensions, except support from significant others, with insomnia were observed at all time points, with the strength of the association being greater in the case of social support from friends (T1 = −0.311, T2 = −0.195, T3 = −0.254).

With regard to self-efficacy, there was a statistically significant and negative relationship with insomnia evaluated at the three time points (*p* < 0.001). Resilience also had a statistically significant and negative relationship with insomnia assessed throughout this study (*p* < 0.001). Finally, cognitive fusion presented the highest correlation (positive values) with insomnia at all time points (*p* < 0.001).

#### 3.4.2. Influence of the Psychosocial Variables on the Evolution of Insomnia through the Three Time Points Considered

Using general linear mixed models, we proceeded to assess the influence of the psychosocial variables considered (as inter-subject factors) on the evolution of insomnia. Each psychosocial variable was transformed into a dichotomous variable (high and low levels), and the cut-off point for our study was the median score of the variable.

**Figure 3:**
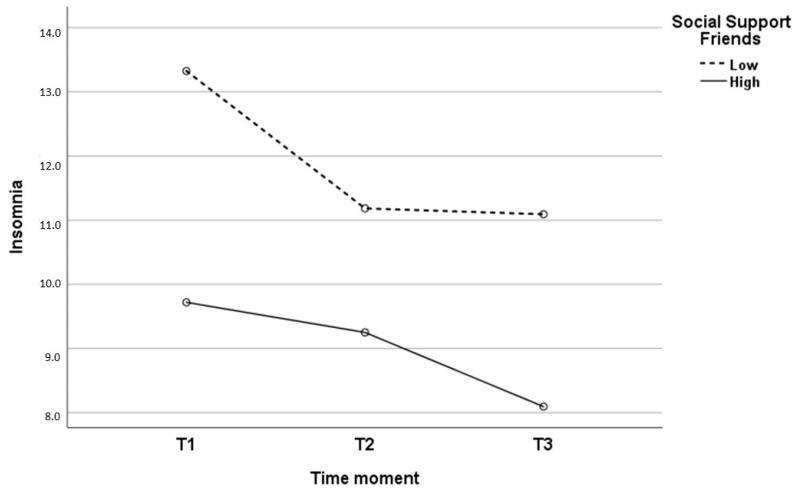
Evolution of insomnia as a function of social support from friends.

**Figure 4:**
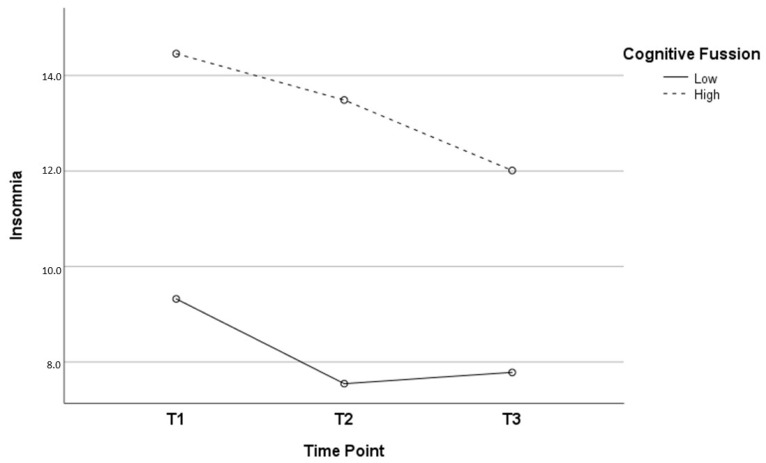
Evolution of insomnia as a function of cognitive fusion.

When analyzing the three time points, two interaction effects were found between the psychosocial variables and the evolution of insomnia, specifically with regard to the social support of friends (*p* = 0.024) and cognitive fusion (*p* = 0.047). The effect size for the interaction between insomnia and social support of friends was ƞ2 = 0.34, and for the interaction between insomnia and cognitive fusion was ƞ2 = 0.25. As can be seen in Table 4 and Figure 3, HCWs with low social support from friends had higher insomnia scores at all three time points. Regarding the evolution of insomnia, the interaction results followed a different course in the two groups (low and high social support). While in the low social support group, significant decreases were observed between T1 and T2, and between T1 and T3, the high social support group hardly presented change in the first two time points, although statistically significant differences were observed in the long term. In relation to cognitive fusion, as can be seen in Table 5 and Figure 4, HCWs with high cognitive fusion had higher insomnia scores at all three time points. In the low cognitive fusion group, significant decreases were found between T1 and T2 (i.e., in the short term) and between T1 and T3. However, decreases in insomnia in the high cognitive fusion group were observed over the longer term (specifically between T2 and T3, and between T1 and T3).

## 4. Discussion

Insomnia poses a significant health problem that directly affects HCWs. Previous research has suggested that insomnia in HCWs is closely related to shift work, especially night shifts, to the fear of making medication errors, to the development of healthcare activity in highly complex units and to the emotional burden inherent to healthcare [14,38]. Furthermore, insomnia in HCWs has been associated with a decrease in the quality of patient care and an increase in medical errors, underscoring the urgent need to address this issue [39].

Many authors have explored the consequences of the pandemic on HCWs and have reported a high prevalence of negative symptoms such as anxiety, depression and stress [40,41,42]. Research has shown that the prevalence of insomnia in HCWs increased during this time, and several circumstances have been identified as risk factors, most notably working with infectious patients, increased workload and fear of contagion [18,40]. However, different studies have suggested that despite the fact that during the pandemic, HCWs suffered a high prevalence of psychoemotional symptoms, insomnia has been the symptom with the highest prevalence, affecting more than half of the professionals in most of the studies [43]. In spite of this finding, most studies conducted to understand and address insomnia in this context have only explored its prevalence at specific points in time, the most frequently studied being the onset of the pandemic and the confinement phases [12], while few have analyzed its evolution over time [44,45].

With the above in mind, it is particularly relevant to analyze the evolution of insomnia in HCWs in order to understand the mid- and long-term emotional impact of the COVID-19 pandemic, as well as to analyze the possible risks and protective variables involved in both its prevention and chronification. Given this need, the aim of our study is to explore the evolution of insomnia in Spanish HCWs, including the analysis of the influence of sociodemographic, occupational and psychosocial variables, both at specific points in the pandemic (early, medium and long term) and throughout the evolution of the pandemic.

Our results suggest a positive trend in the evolution of insomnia among HCWs two years after the onset of the COVID-19 pandemic, recording a significant positive evolution between T1 and T2 and a non-significant evolution between T2 and T3. This finding is consistent with the results of previous studies that have shown a significant reduction in the prevalence of insomnia in HCWs one year after the onset of the pandemic [46]; however, not all studies have found this, with some reporting a higher prevalence of insomnia during the second time point [47].

In the analysis of the possible sociodemographic and occupational variables that influence insomnia, our study shows that professional category has a direct and significant relationship with insomnia during the first two time points, with nurses having the highest mean values for insomnia. Recent findings suggest that insomnia affects nursing professionals to a greater extent [23], as it is often associated with night work, fear of making medication errors, work performance in highly complex units, and the emotional burden inherent to health care [48]. Research conducted at the beginning of the COVID-19 pandemic states that working directly with infectious patients is considered a risk factor associated with high levels of anxiety and insomnia [18].

Our results found that there is a significant relationship between insomnia and the family situation of HWCs at the first time point, with higher means for HCWs who had a partner. This is in accordance with previous research during the COVID-19 pandemic that reported a direct association between being married and depression or insomnia [49]. It should be noted that the first time point in our study was during the confinement phase of the pandemic, and during this period, findings were found linking cohabitation with a partner with anxiety [50].

Regarding occupational variables, our findings suggest that work overload experienced by HCWs throughout the COVID-19 pandemic had a significant direct relationship with insomnia at all three time points. These results support others found in the literature that associate a high perception of workload with high levels of insomnia [51]. Likewise, we found that the lack of PPE was significantly related to insomnia during the first time point, similarly to what was found in a Chinese HCWs sample at the beginning of the pandemic [52]. Concerns about self-contagion or infection of a family member has been one of the most studied risk factors during the COVID-19 pandemic [51,53,54]. In our study, it is also presented as a risk factor for insomnia during the three time points, showing a significant association with insomnia. Regarding the service in which the care task is carried out, despite the existence of several studies showing that professionals working in certain types of units (ICU, emergency room) have higher levels of anxiety [55,56], we did not find this relationship in the case of insomnia.

One of the most novel aspects of our study is the consideration of psychosocial variables. In particular, bivariate analyses show social support as a protective variable for insomnia. In this case, social support derived from friends and family was particularly important, maintaining significantly negative relationships at all three time points, holding true for total social support. These results suggest that social support plays a fundamental role in the prevention and management of insomnia among HCWs. Recent research has shown a significant negative association between social support and insomnia in HCWs [57]. Having a strong support system, both inside and outside the work environment, has been identified as a crucial protective factor against the development and perpetuation of insomnia [58]. Specifically, the importance of social support derived from friends in stressful situations has become clear, acting as a buffer against negative psychoemotional consequences [59].

In the analysis of the effect of self-efficacy, we found that it behaves as a clear protector against insomnia and in a sustained manner over time, buffering the effects of being in stressful work situations. Recent research has found an association between self-efficacy and insomnia in the general population, in line with our results [60,61]. Meanwhile, specifically among nurses it has been found that in stressful work situations, self-efficacy acts as a clear protector in the management of anxiety and can even have positive consequences, such as the development of a resilient personality, if the anxiety derived from stressful work situations is managed through self-efficacy [62].

Resilience has been one of the most studied traits during the COVID-19 pandemic in HCWs [63,64]. Our findings show significant results that highlight the negative association between resilience and insomnia during the three time points of analysis. These findings corroborate the importance of resilience as a protective factor against insomnia in HCWs, further affirming that its protective effect is maintained over time. Resilience, understood as the ability to adapt positively to adversity, has been shown to be a crucial factor in the prevention and management of insomnia in this field [65,66,67].

Throughout the COVID-19 pandemic, cognitive fusion, characterized as an over-identification with thoughts and an inability to distract oneself from them, has been found to be a clear precipitator of negative psychoemotional consequences on HCWs [41]. Its relationship to consequences of work-related stressors, such as anxiety, has also been previously studied [24]. Our results, in the absence of previous studies, show its role as a risk factor for insomnia, increasing symptoms at short-, medium- and long-term stages of the COVID-19 pandemic and affecting its evolution. Regarding its influence on the development of insomnia, HCWs with low cognitive fusion experienced improvements in insomnia in the short term (six months after the most critical phase of the COVID-19 pandemic), proving it to be a very valuable personal resource for coping with highly stressful situations. Given the absence of prior research on the matter, our study highlights the importance of addressing cognitive fusion in the prevention and treatment of insomnia in order to establish strategies aimed at mitigating its impact on HCWs. Different techniques based on Acceptance and Commitment Therapy have shown their effectiveness in reducing this risk factor [68].

Given the longitudinal nature of our study, and in the absence of previous studies evaluating the evolution of insomnia in relation to psychosocial variables, a very novel finding our results show is that both social support from friends and cognitive fusion are variables that affect the evolution of insomnia over the time period considered. In particular, social support from friends is shown to be a clearly positive variable in the long-term course of insomnia, as HCWs with high social support experienced significantly less insomnia at the three time points assessed, with no major variations in the mid-term (six months after the most critical phase of the COVID-19 pandemic) and with a significantly favorable evolution in the long term. We must not forget that the early phases of the pandemic were characterized by clear restrictions on social relationships, which could justify the significant effect of social support on long-term insomnia. Regarding cognitive fusion, as noted above, our results indicate that ‘defusing’ thoughts is shown to be a useful short-term resource for coping with highly stressful situations.

Finally, it is necessary to point out some of the limitations of our research. Among them, we can highlight the non-probabilistic convenience sampling, which limits the generalization of the results. The low participation of males may lead to a bias in terms of gender analysis, although this low representation corresponds to the reality of the profession in Spain. In addition, to find out the real impact of the COVID-19 pandemic on insomnia, it would have been of interest to have previous (baseline) assessments of the participants’ insomnia from before the pandemic. On the other hand, although the present research focuses specifically on insomnia and its evolution, it would have been interesting to contextualize insomnia symptoms within the stress experienced and the associated “Dysregulation of Mood, Energy, and Social Rhythms Syndrome” (DYMERS). This syndrome is characterized by a poor regulation of biological, social and behavioral rhythms, including nutrition, social contacts and sleep [69]. In fact, this syndrome was the ground of vulnerability for various mental health problems during the COVID-19 pandemic, including panic disorder, attention deficit hyperactivity disorder and post-traumatic stress disorder [70]. Future lines of research could go further in this direction as far as insomnia is concerned, assessing both insomnia and other mental health pathologies, with a special interest in DYMERS, and their impact on quality of life and psychological well-being.

## 5. Conclusions

Several studies highlight that HCWs have higher rates of insomnia compared to the general population [51], which can compromise both their personal well-being and their ability to provide quality care. In addition, insomnia in HCWs has been associated with a decrease in the quality of patient care and an increase in medical errors, underscoring the urgent need to address this problem.

Our study shows not only the evolution of insomnia over three time periods and its association with different sociodemographic and occupational factors, but also its relationship with different psychosocial variables, contributing a new line of research to the existing literature. Of particular interest are the roles of social support and cognitive fusion in the evolution of insomnia, highlighting the importance of taking these variables into account in the evaluation of risk factors and in the creation of appropriate intervention programs for HCWs.

## Figures and Tables

**Table 1:** Relationship of insomnia assessed at the three time points with sociodemographic and occupational variables.

				Insomnia								
				Time Point 1			Time Point 2			Time Point 3		
		f (%)	Mean (SD)	Mean (SD)	r	*p*	Mean (SD)	r	*p*	Mean (SD)	r	*p*
Age			42.89 (9.84)		−0.040	0.555		0.042	0.496		0.114	0.067
Experience			11.06 (9.29)		0.051	0.456		0.109	0.081		0.089	0.151
Gender	Men	34 (15.7%)		9.88 (6.66)		0.050	8.65 (6.38)		0.087	9.44 (6.98)		0.741
	Women	182 (84.3%)		12.06 (5.78)			10.64 (6.16)			9.81 (5.91)		
Job category	Nurse	151 (69.9%)		12.64 (5.95)		0.001	10.90 (6.17)		0.038	10.14 (6.25)		0.162
	Physician	65 (30.1%)		9.60 (5.48)			8.99 (6.19)			8.88 (5.60)		
Cohabitation	Without a partner	66 (30.6%)		10.49 (5.79)		0.043	9.27 (6.25)		0.100	8.92 (6.33)		0.181
	With a partner	150 (69.4%)		12.26 (5.98)			10.79 (6.18)			10.13 (5.95)		
Employment situation	Permanent	114 (52.8%)		11.51 (5.99)		0.283	10.51 (6.24)		0.436	10.44 (6.22)		0.340
	Interim	57 (26.4%)		12.90 (6.12)			11.33 (6.31)			10.56 (6.59)		
	Temporary	45 (20.8%)		11.96 (5.75)			9.92 (6.01)			9.06 (6.33)		
Service	ICU	77 (35.6%)		12.47 (6.21)		0.159	10.95 (5.99)		0.566	10.85 (6.27)		0.430
	Hospitalization	59 (27.4%)		11.84 (5.79)			10.59 (6.58)			10.33 (6.99)		
	Primary Care	41 (19.0%)		11.79 (5.37)			11.21 (6.48)			10.27 (5.77)		
	Emergency	29 (13.4%)		12.71 (6.25)			10.27 (5.64)			8.97 (6.17)		
	Others	10 (4.6%)		8.28 (5.37)			8.14 (6.67)			8.07 (5.61)		
Work shift	Shifts (M, A, N)	54 (25.0%)		11.89 (5.66)		0.302	10.80 (6.53)		0.456	10.77 (5.31)		0.685
	Rotating	109 (50.5%)		12.28 (5.99)			10.89 (5.97)			9.94 (6.85)		
	>10 h	53 (24.4%)		11.57 (6.47)			10.01 (6.38)			10.21 (6.42)		
Workload	Equal	36 (16.7%)		8.11 (5.49)		<0.001	6.86 (5.28)		<0.001	7.86 (5.69)		0.040
	Higher	180 (83.3%)		12.44 (5.81)			11.02 (6.18)			10.14 (6.10)		
PPE availability	Yes	90 (41.6%)		10.14 (5.23)		<0.001	9.46 (5.50)		0.083	9.44 (5.84)		0.521
	No	126 (58.4%)		12.85 (6.21)			10.94 (6.65)			9.98 (6.26)		
Worry about contagion	Own		2.70 (0.89)		0.211	0.002		0.186	0.003		0.219	<0.001
	Family		3.54 (0.84)		0.204	0.003		0.240	<0.001		0.209	<0.001

**Table 2:** Distribution of insomnia at the three time points according to the ISI classification.

	T1 n (%)	T2 n (%)	T3 n (%)
**No clinically significant insomnia** 0–7 *	54 (25.0%)	73 (33.8%)	77 (35.6%)
**Subthreshold insomnia** 8–14 *	90 (41.7%)	89 (41.2%)	90 (41.7%)
**Clinical insomnia (moderate severity**) 15–21 *	59 (27.3%)	45 (20.8%)	44 (20.4%)
**Clinical insomnia (severe)** 22–28 *	13 (6.0%)	9 (4.2%)	5 (2.3%)

* Classification of insomnia levels.

**Table 3:** Descriptives of psychosocial variables and their association with insomnia at the three time points.

		Insomnia					
		T1		T2		T3	
	Mean (SD)	*R* ^2^	*p*	*R* ^2^	*p*	*R* ^2^	*p*
SS^1^ family	5.88 (1.20)	−0.167	0.007	−0.164	0.008	−0.151	0.015
SS^1^ friends	5.64 (1.39)	−0.311	0.001	−0.195	0.002	−0.254	<0.001
SS^1^ other	5.81 (1.55)	−0.083	0.182	−0.026	0.675	−0.054	0.390
SS^1^ total	5.78 (1.21)	−0.211	0.014	−0.141	0.024	−0.171	0.006
Self-efficacy	29.18 (4.07)	−0.275	<0.001	−0.282	<0.001	−0.305	<0.001
Resilience	78.39 (14.26)	−0.261	<0.001	−0.226	<0.001	−0.244	<0.001
Cognitive Fusion	21.95 (10.80)	0.454	<0.001	0.558	<0.001	0.370	<0.001

SS^1^: Social support.

**Table 4:** Evolution of insomnia in HCWs with low and high social support from friends.

	Social Support Friends			
	Low	High		
	Mean (SD)	Mean (SD)	t	*p*
Insomnia T1 (1)	13.32 (5.94)	9.71 (5.38)	4.798	<0.001
Insomnia T2 (2)	11.18 (6.44)	9.25 (5.78)	2.659	0.008
Insomnia T3 (3)	11.09 (6.12)	8.09 (5.62)	3.970	<0.001
	F = 14.338 *p* < 0.001 (1/2; 1/3)	F = 5.902 *p* = 0.003 (1/3; 2/3)		

**Table 5:** Evolution of insomnia in HCWs with low and high cognitive fusion.

	Cognitive Fusion			
	Low	High		
	Mean (SD)	Mean (SD)	t	*p*
Insomnia T1 (1)	9.32 (5.35)	14.45 (5.45)	−7.232	<0.001
Insomnia T2 (2)	7.54 (5.43)	13.48 (5.53)	−8.627	<0.001
Insomnia T3 (3)	7.78 (5.43)	12.01 (6.01)	−6.146	<0.001
	F = 11.974 *p* < 0.001 (1/2; 1/3)	F = 8.570 *p* < 0.001 (1/3; 2/3)		

## Data Availability

Research data will be available upon request to the corresponding author.
